# Transcranial ultrasound localization microscopy in moyamoya patients using a clinical ultrasound system

**DOI:** 10.7150/thno.105427

**Published:** 2025-03-10

**Authors:** Louise Denis, Elena Meseguer, Augustin Gaudemer, Georges Jaklh, Sylvain Bodard, Georges Chabouh, Dominique Hervé, Eric Vicaut, Pierre Amarenco, Olivier Couture

**Affiliations:** 1 Sorbonne Université, CNRS, INSERM Laboratoire d'Imagerie Biomédicale, Paris, France.; 2 Department of Neurology Bichat University Hospital (APHP), Paris, France.; 3 Clinical Research Unit Lariboisière-Fernand Vidal (APHP), Paris, France.; 4 CERVCO, Lariboisière Hospital, (APHP Nord), Paris, France.; 5 Center for Transplantation Sciences, Massachusetts General Hospital, Harvard Medical School, Boston, Massachusetts, USA.; 6 AP-HP, Hôpital Necker Enfants Malades, Service d'Imagerie Adulte, F-75015, Paris, France.; 7 Université de Paris Cité, Paris, France.

**Keywords:** ULM, transcranial, ultrasound, super-resolution, moyamoya

## Abstract

**Background:** Deep brain structures are supplied by perforating arteries, which are too thin to be observed with non-invasive and widely available clinical imaging methods. In moyamoya disease, main arteries in the base of the brain progressively narrow, and perforating arteries grow densely and tortuously to compensate the lack of blood supply in deep brain structures.

**Purpose:** The aim of this study is to evaluate the efficacy of transcranial ultrasound localization microscopy (ULM) in visualizing perforating arteries, utilizing a standard low-frame-rate ultrasound clinical scanner and contrast sequences commonly employed in hospital settings.

**Methods:** This prospective single-center study included ischemic stroke patients not related to the study of perforating arteries, and moyamoya disease patients. Contrast-enhanced ultrasound sequences (CEUS) were performed by an experienced neurologist and the images acquired were used to perform post-processing ULM. ULM density maps were compared with conventional 3T TOF MRI and color Doppler imaging in both groups.

**Results:** We included a group of 15 control patients and another group of 9 moyamoya patients between March 2023 and March 2024. The patients had an average age of 45 ± 14 years (65% male). Perforating arteries were captured on all subjects, with a mean diameter of 0.8 ± 0.3 mm in control patients, while it was not possible with TOF MRI or color Doppler (P < 0.05). Moreover, ULM enabled to highlight differences between healthy subjects and those with moyamoya disease through track mean distance (P = 0.05).

**Conclusions:** Using a low-frame-rate ultrasound scanner, CEUS and accessible post-processing tools, we demonstrate that transcranial ULM can facilitate the visualization and characterization of perforating arteries, even in cases where they were previously undetectable using standard non-invasive imaging techniques. We speculate that with the advent of high-frame-rate 3D ULM, this technique may find widespread utility in hospitals.

## Introduction

Perforating arteries originate from the anterior choroidal artery (lenticulostriate, LSAs), the anterior cerebral artery (ACA) or the middle cerebral artery (MCA). Ranging from 0.1 to 1.2 mm in diameter 1, they are responsible for the blood supply to deep brain territories 2. Perforating arteries are involved in moyamoya disease, a rare chronic arteriopathy that arises from an occlusion, partial or complete, of the carotid artery inside the skull. Terminal carotid progressive stenosis promotes the development of an additional artery network to ensure cerebral perfusion. As a result, perforating arteries develop through various neo-anastomosis strategies 3.

Currently, arteriography of perforating arteries is used as the standard to establish the different stages of moyamoya disease, but it remains an invasive and ionizing technique 4. 7T TOF MRI has also proven effective in imaging perforating arteries, including in patients with moyamoya symptoms 5. However, these high-field devices are not widely available in hospitals, and lower-field (1.5-3T) TOF MRIs are limited in spatial resolution in the visualization of perforating arteries. Transcranial ultrasound (US) is routinely used in clinical practice for non-invasive assessments. While color Doppler sequences enable visualization of the Circle of Willis and its communicating arteries, they are limited in detecting perforating arteries due to ultrasound wave diffraction 6. Contrast-enhanced ultrasound (CEUS) techniques facilitate perfusion measurements to evaluate the underlying conditions of perforating arteries, but do not provide their direct visualization 7,8,9.

Ultrasound localization microscopy (ULM) has recently proved effective in visualizing microscopic vessels in deeper regions using US contrast agents and high frame rate sequences 10,11,12,13,14. Volumetric ULM has been used to highlight cerebral micro vessels 15,16,17,18,19,20,21,22, including ischemic areas in rodents 23. Transcranial 2D ULM has also been used to observe a case of cerebral aneurysm and a case of moyamoya in humans 24. However, the application of transcranial 2D ULM in humans has been hindered by the requirement of a high frame rate US scanner, which is not commonly accessible in hospital settings.

The aim of this study is therefore to extend the viability of low frame rate 2D ULM 25,26,27 for the observation and differentiation of cerebral perforating arteries between control patients without involvement of perforating arteries and patients with moyamoya symptoms. By using conventional sequences (CEUS) and accessible tools 28, this study aims to accelerate the clinical transfer of ULM to ultrasound scanners already available in hospitals.

## Materials and Methods

### Ethical approval

This study received ethics committee approval in March 2023 (national n° 2022-A02486-37). Each patient gave an informed consent for his/her participation in the study.

### Population study

This prospective, single-center, non-interventional study included 24 patients from March 2023 to March 2024. A first group of 9 patients with moyamoya disease was selected by an expert center for this pathology (4 women and 5 men). A second group of 15 ischemic stroke patients, not related to perforating arteries, as confirmed with MRI, i.e., control patients, was included (3 women and 12 men).

**Figure 1:**
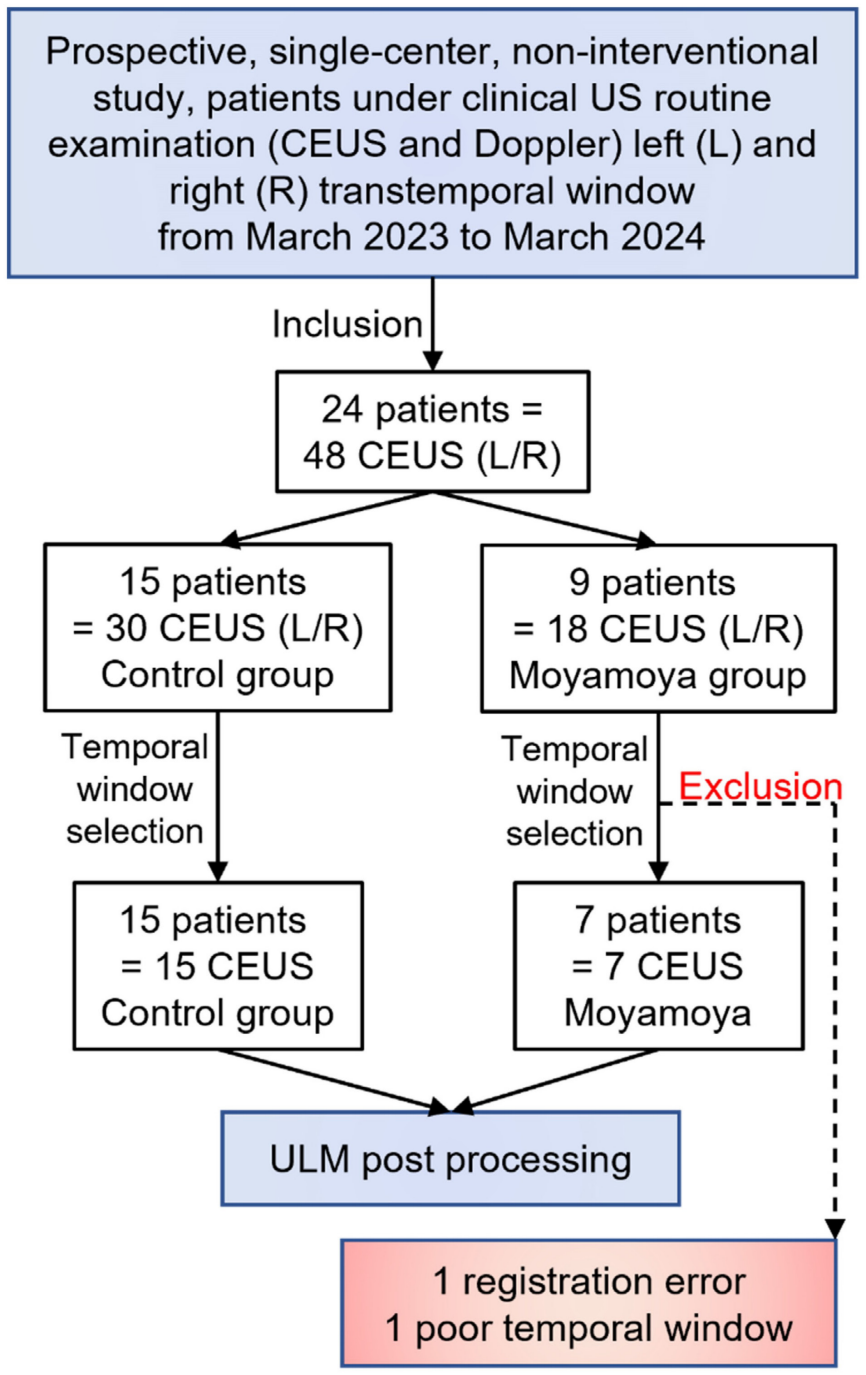
** Study population.** 24 patients were included, with 15 of them from a control group and 9 others with Moyamoya symptoms.

All patients underwent a clinical routine US examination, including color Doppler and CEUS sequences. Based on the patients' temporal window and motion estimation, 21 CEUS acquisitions in total were selected for ULM post-processing, i.e., 3 moyamoya patients were excluded due to poor registration related to motion and a poor temporal window, as assessed by preliminary transcranial Doppler (Figure 1, Supplementary Methods). Table S1 summarizes patient characteristics, and Table S2 summarizes CEUS selection.

### US acquisitions

Acquisitions were performed using a clinical US scanner (Siemens Acuson S2000) and a commercial US probe (M4v1c, 2 MHz) handheld by the clinician. For each patient, an initial color Doppler sequence was acquired before US contrast agent injection, to establish the quality of the acoustic window and ensure probe positioning in the MCA region (Supplementary Methods). CEUS mode was then used, as in clinical routine, alongside an intravenous injection of a 2 ml bolus of Sonovue (Bracco, Italy) separated in one or two injections, and followed by an injection of 10 cc of serum physiology (Supplementary Figure S1). CEUS clips were manually saved, and the total acquisition lasted from 31 to 230 seconds per patient. The variations in clip length were due to the manual clip recording and manual clip selection, i.e., in some patients, only one clip was used to perform ULM post-processing, whereas in other patients several clips were selected as there was no movement (Figure S2). At the end of the bolus, a new color Doppler image was acquired, with the remaining contrast agent. The imaging depth was 9 cm, the imaging rate was 19 Hz, and the clips lasted around twenty seconds each. The initial pixel resolution was 0.13 mm. The non-derated mechanical index was 0.6, and the cranial thermal index was 1.6 (in CEUS mode, Figure S1). CEUS clips, i.e., screen capture from the ultrasound scanner, were anonymized and exported in TIFF format. The data were then post-processed in the laboratory using MATLAB 2020a.

### ULM post-processing steps

CEUS clips were used to perform ULM density maps, i.e., number of microbubbles tracked per pixel, thanks to several conventional steps 11,28. First, we estimated movement by estimating cross-correlation between Power Doppler of the first clip, i.e., the sum of all frames contained in the CEUS clip with a 2-15 Hz bandpass filter, and Power Doppler of the other clips (Supplementary Figure S2). We also estimated concentration by displaying intensity as a function of time, i.e., the duration of the CEUS clips (Supplementary Figure S2). Upon these measurements, we selected optimal CEUS clips, i.e., with no movement and distinguishable microbubbles (see Supplementary Figure S2). A filtering step was then performed by applying time gain compensation for signal attenuation at depth. A binary perfusion mask was also applied to reduce the probability of noise localization (Supplementary Figure, S3).

**Figure 2:**
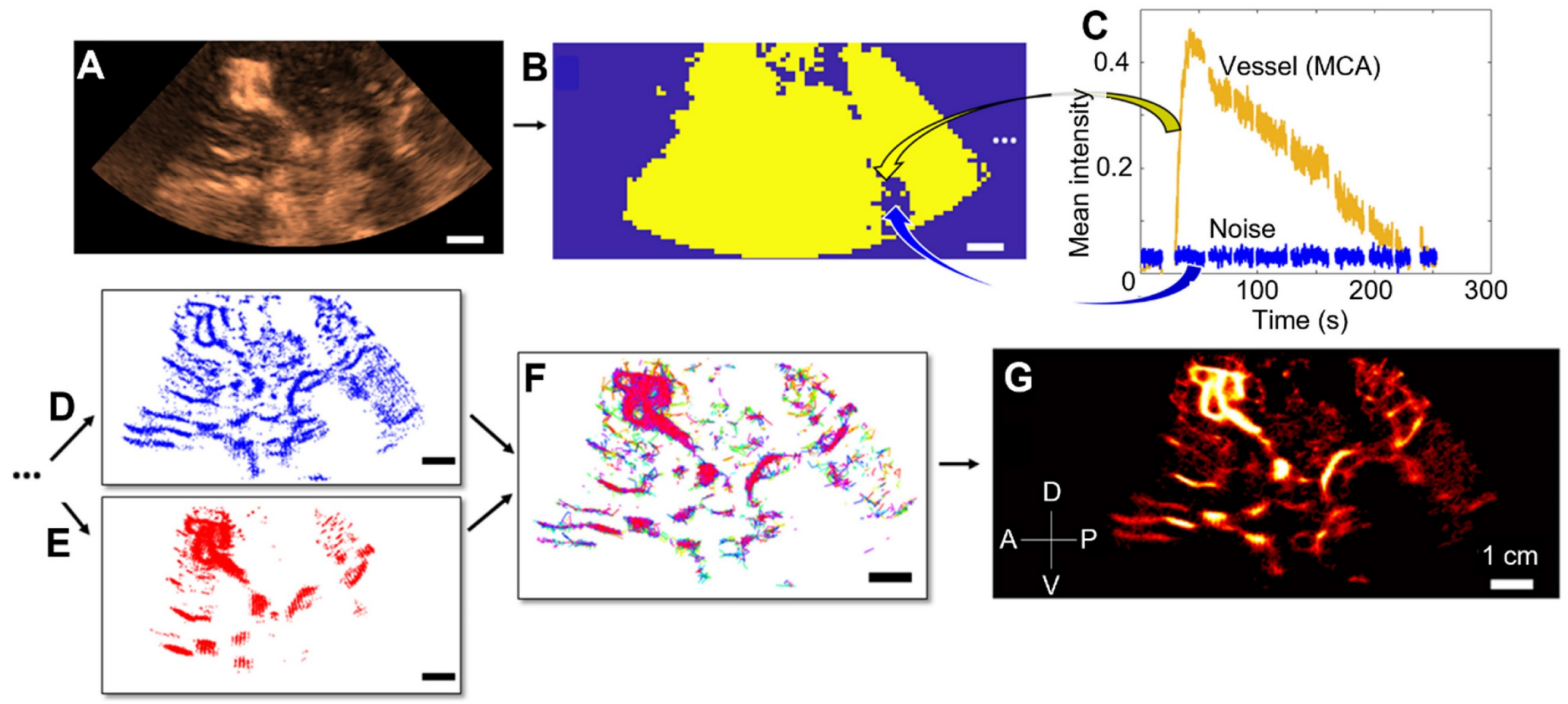
** Steps of 2D transcranial ULM, example on patient n°1.** A) Acquisition of clips, sorting of images with high noise level or large motion (SF1) and adjusting the TGC settings, B) Estimation of the binary perfusion mask (SF2), with typical MCA and noise patterns highlighted in C), D) Localization of concentrated MB by 10-pixel Gaussian correlation, E) Localization of isolated MB by 30-pixel Gaussian correlation, F) Tracking step with merged localization of MB, G) Accumulation of the tracks and reconstruction of a density map. Same scale everywhere: 1 cm.

Microbubbles were then localized by Gaussian correlation, i.e., normalized cross-correlation with a PSF (Point Spread Function) of two different sizes. The Point Spread Function (PSF) was simulated at the center of a fixed-edge kernel, and we adapted the edge of the kernel and the standard deviation σ of the PSF to localize both concentrated and isolated microbubbles. For concentrated microbubbles, the PSF was set to an empiric value of 10 pixels in edge length, hence two microbubbles are separated by a distance of 2λ 28,29. Note that pixel size was λ/5. For isolated microbubbles, the PSF was set to 30 edge pixels, i.e., a spacing of 5λ between each microbubble, to include more isolated microbubbles. We also adjusted the standard deviation (σ) of these two PSFs to accommodate the widening of the PSF at depth, expanding from λ at the surface to 3λ at depth. Further details regarding the impact of PSF size and σ can be found in the Supplementary Materials (Figures S4, S5, S6). All the microbubbles localized were then merged (while being counted only once), and tracked using the Hungarian algorithm with a distance between microbubbles from 2λ to 3λ and a minimum duration of 0.1 s 30,31, with a gap of 0.05 s allowed (Table S3). Thus, our system can track a speed of 45.9 mm/s at maximum. If the object moves more slowly than this, it can still be reliably tracked by the algorithm. The tracks were interpolated with a factor of λ/10 and they were then accumulated and projected onto a final grid, i.e., a pixel size of λ/5. A comprehensive overview of all the ULM steps is provided in Figure 2.

### Image analysis

To estimate vessel diameters on the ULM density map, cross-sections of the vessels of interest were manually segmented. This segmentation was done after registration of ULM on MRI by the radiologist to be sure of the global positioning of the perforating arteries ROI. The perforating arteries cross-section is the result of an arbitrary choice of the most visible arteries on the image. Intensity was plotted as a function of spatial distribution along the section, followed by the measurement of Full Width at Half Maximum (FWHM) 11,24.

Color Doppler acquisitions were taken from the same plane as the CEUS sequences. The 3T TOF MRI, with maximum intensity projected over 4 mm of thickness, was aligned on the ULM mapping by a neuroradiologist (CareStream software). The same plane between the ULM and the TOF 3T MRI was manually achieved through multiplanar reformatting, taking the same angulation as that used for the color Doppler and CEUS, starting with the location of the carotid arteries on the image. The MCA region was manually segmented to compare the vessel density, i.e., the number of pixels above the image intensity median, in each technique.

A comparison of perforating arteries reconstructed by ULM density between control patients and moyamoya pathology patients was carried out in the same MCA regions as those previously manually segmented, i.e., for comparison of imaging techniques. Thus, in the MCA areas, we could estimate the number of microbubbles localized, the number of tracks accumulated, the number of perforating arteries, i.e., more than 8 pixels connected, and the spatial distribution of the tracks, i.e., mean distance. This mean distance, also called spatial distribution, is measured as the average of the distances between each point on the track and the centroid of all the tracks, i.e., center of mass, within the perforating arteries ROI. All these metrics have been measured on interpolated tracks, with the same ULM parameters on all patients (Supplementary Table S3).

### Statistical analysis

One-way ANOVA tests were used to compare ULM, TOF MRI, and color Doppler vessels' density (Figure 4 and 5). We used unpaired two-sided parametric Student's t-tests with a 95% confidence level assuming a Gaussian distribution (Kolmogorov-Smirnov test) to compare the two groups, PSF size and σ (Figure 6 G-H and S4). When the distribution was not normal, we used the Mann-Whitney test (Figure 6 E-F). All statistics were performed on GraphPad Prism 9 software.

## Results

### Patient characteristics

The average age of the 15 patients in the control group was 47 ± 14 years, and 80% were male (Table S1). The right temporal window was chosen in 9/15 cases and CEUS clips ranged from 46 to 182 seconds (Table S2).

In the moyamoya group, the mean age was 43 ± 15 years, and the chosen time window for included patients was the right temporal one in 3 out of 6 cases (Table S1). Patients were predominantly male (5 out of 6 cases), and CEUS clips ranged from a minimum of 31 s to a maximum of 230 s (Table S2).

All these demographic characteristics, summarized in Tables S1 and S2, suggest that the two groups are comparable. In the moyamoya group, the development of perforating arteries was bilateral in 3 out of 9 cases.

### ULM density maps allow the reconstruction of perforating arteries in the control group

**Figure 3:**
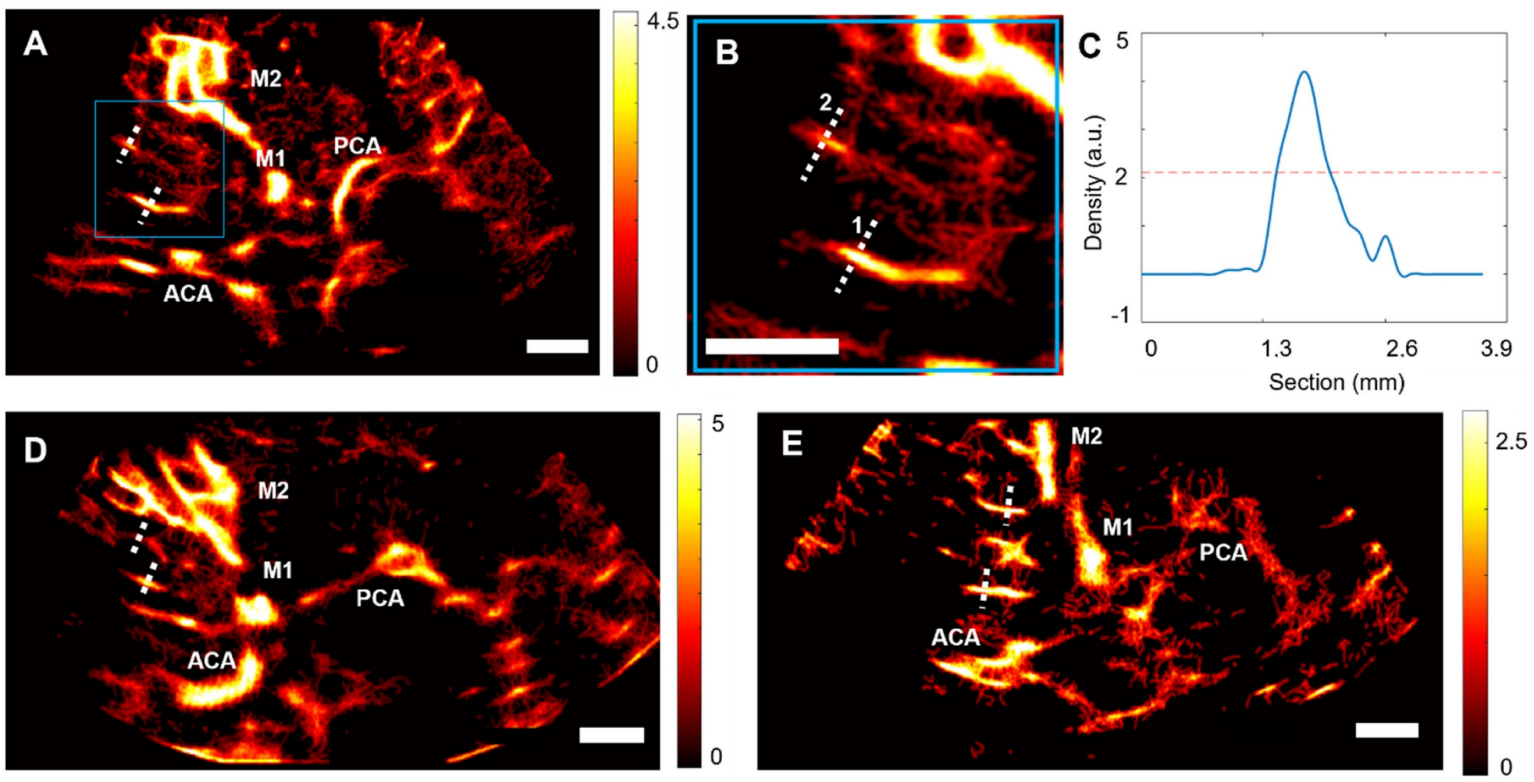
** ULM density maps on control patients**. A) ULM density map on patient 1, right temporal window. White dotted lines indicate segmented vessels. The blue square represents the zoomed area in B), C) Density as a function of the lateral cross-section of the first segmented vessel. The full width at half maximum (FWHM) corresponds to the vessel diameter, i.e. 0.6 mm here. D) ULM density map on patient 8, left temporal window. White dotted lines indicate segmented vessels. E) ULM density map on patient 10, left temporal window. White dotted lines indicate segmented vessels. All color bars represent the density, i.e. count of microbubbles per pixel (in the arbitrary unit, a.u.). Scale bars of 1 cm. M1: sphenoidal segment of the MCA (Middle Cerebral Artery), M2: insular segment of the MCA, ACA: Anterior Cerebral Artery, PCA: Posterior Cerebral Artery.

Using post-processing tools on CEUS acquisitions at low frame rates, ULM density maps could be reconstructed in the 15 control patients. The ULM localization error, as defined in 32,33, was measured at 168 ± 12 µm. In addition to allowing the observation of large vessels, such as the MCA and the Circle of Willis, we were able to observe perforating arteries in the region of the MCA (Figure 3A, as shown in the zoomed-in Figure 3B).

In each of the patients in the control group, we segmented two perforating arteries and studied the diameter of these vessels using a calculation of the full width at half maximum (FWHM) of the intensity (see methods) (Figure 3C-E). On average, the segmented perforating arteries had a width of 0.8 ± 0.2 mm, which corresponds to the size observed in the literature, i.e., from 0.1 to 1.2 mm in diameter 1. The comparison between the segmentation of one perforating artery in CEUS acquisition, Power Doppler, i.e., temporal sum of all images in clips, and ULM density map (Supplementary Figure S7) confirmed that ULM allows for finer vessel reconstruction than classical contrast mode.

### Comparison of ULM with TOF 3T MRI and color Doppler in the control group

**Figure 4:**
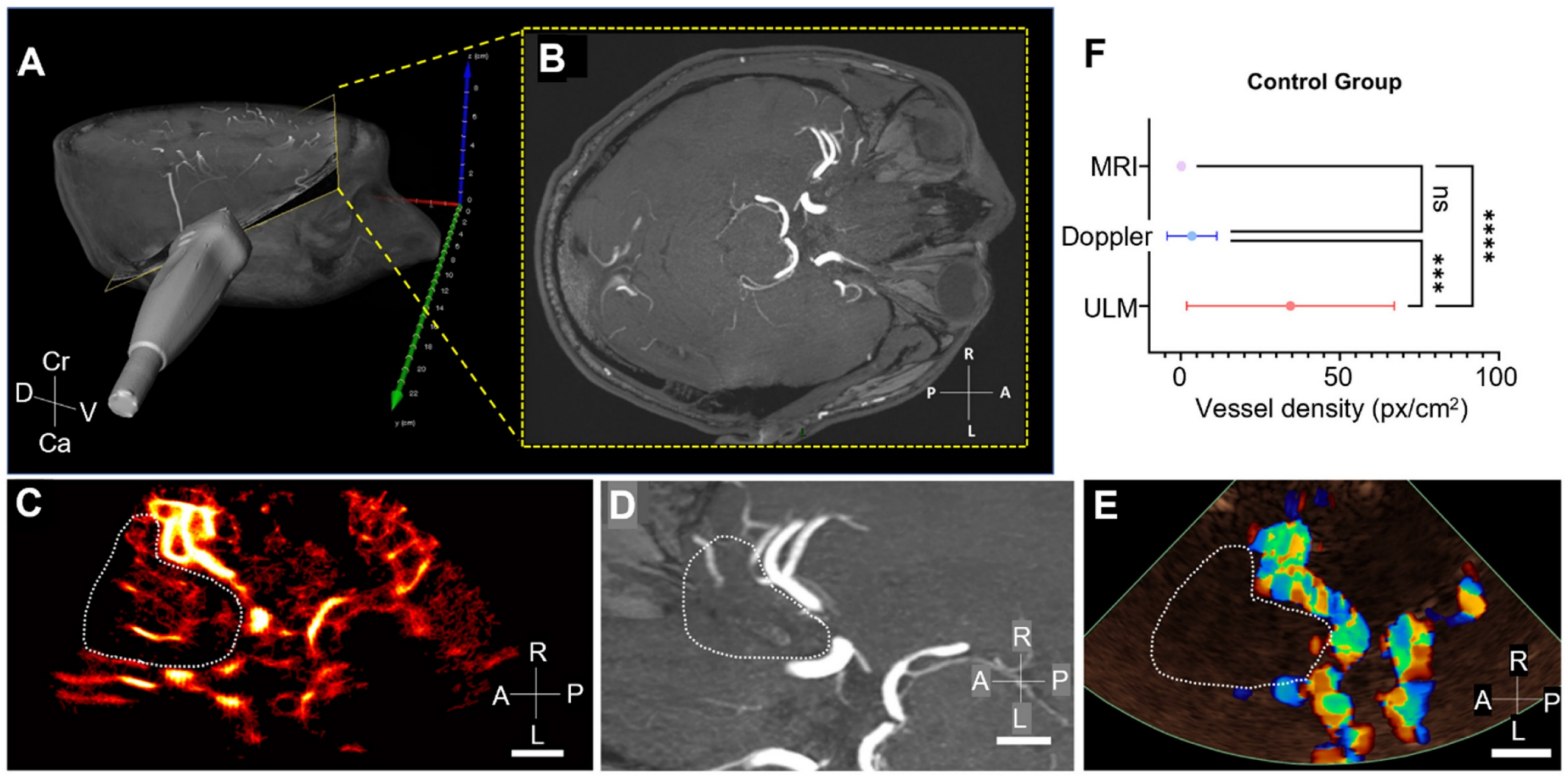
** Comparison of vessel density in MRI, ULM, and color Doppler in control patients**. A) Position of the phased-array ultrasound probe on the right temporal window in patient 1. Probe is superimposed on a 3D TOF MRI volume in a profile view, B) the yellow-framed slice corresponds to the registered TOF MRI slice in an axial view. C) ULM density map on the first patient (control group). White dotted lines indicate perforating arteries' area segmentation, D) MRI TOF 3T of the same patient registered in b). White dotted lines indicate perforating arteries' area segmentation, E) Color doppler of the same patient. White dotted lines indicate perforating arteries' area segmentation, F) Comparison of vessel density, i.e. number of pixels superior to the median intensity of the image, between the three techniques. One-way ANOVA test (see Methods). Scale bars of 1 cm. Radiological notation, D: Dorsal, V: Ventral, Cr: Cranial, Ca: Caudal, P: Posterior, A: Anterior, R: Right, L: Left.

The ultrasound slice plan used to perform CEUS, i.e., ULM, and color Doppler acquisitions could be manually retrieved from the 3T TOF MRI (Figures 4A-B). Thus, we were able to compare the MCA region of the same slice in ULM (Figure 4C), TOF MRI (Figure 4D), and color Doppler (Figure 4E).

Overall, vessel density, calculated as the number of pixels greater than the median intensity of the image (see section Methods), is greater in ULM than in MRI and color Doppler in the MCA region in all 15 patients. An example of vessel density counting is shown in patient 1 (Supplementary Figure S8). Remarkably, the ULM density map enables reconstruction of perforating arteries that are not visible with color doppler and 3T TOF MRI used in clinical routine.

### Comparison of ULM with 3T TOF MRI and color Doppler in the moyamoya group

**Figure 5:**
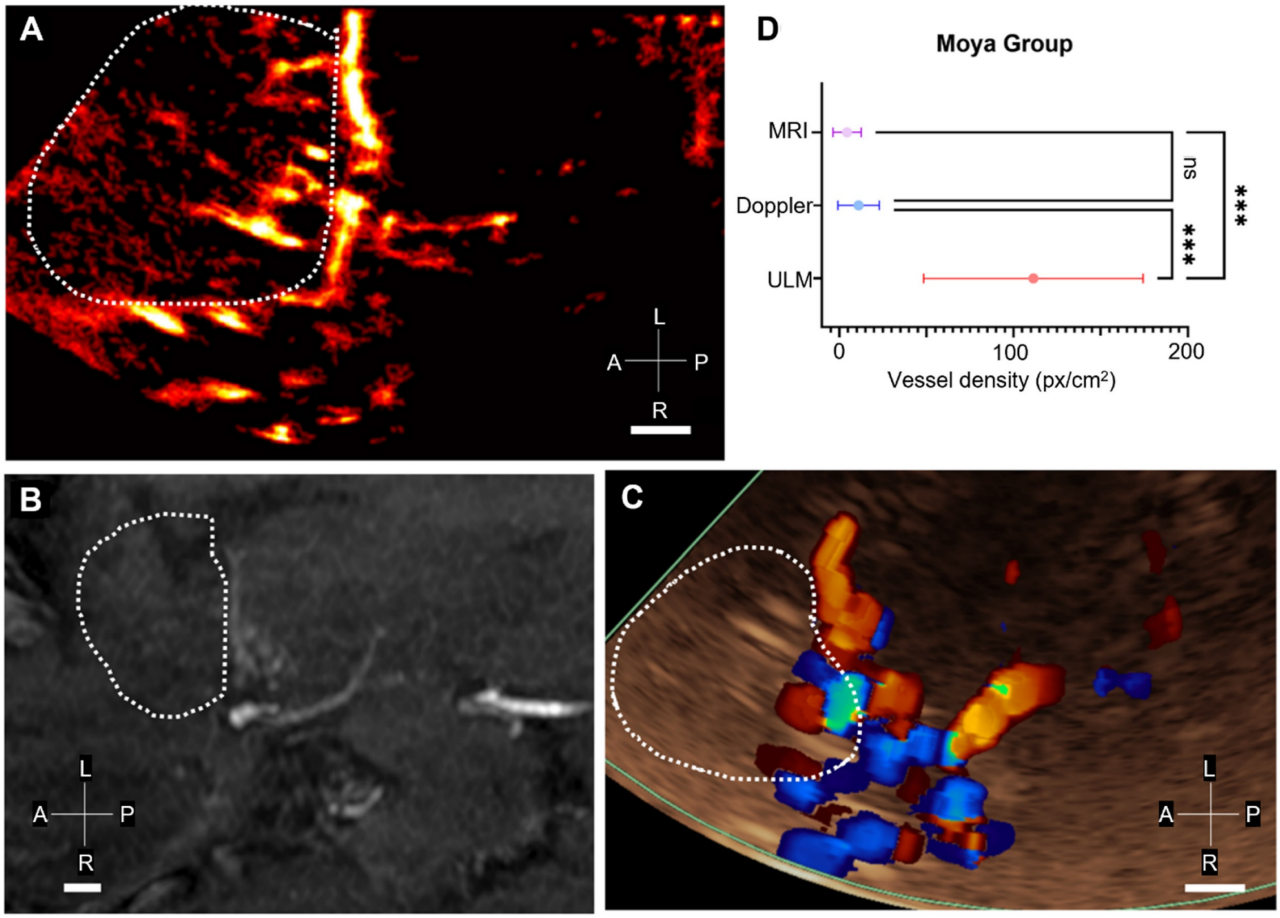
** Comparison of vessel density in MRI, ULM, and color Doppler in Moyamoya patients**. A) ULM density map on the patient n°16 (Moyamoya group). White dotted lines indicate perforating arteries' area segmentation, B) MRI TOF 3T of the same patient (axial view). White dotted lines indicate perforating arteries' area segmentation, C) Color doppler of the same patient. White dotted lines indicate perforating arteries' area segmentation, D) Comparison of vessel density, i.e. number of pixels superior to the median intensity of the image, between the three techniques. One-way ANOVA test (see Methods). Scale bars of 1 cm. Radiological notation, P: Posterior, A: Anterior, R: Right, L: Left.

In patients in the moyamoya group, the same 3T TOF MRI registration step was carried out with ULM and color Doppler (Figure 5A-C). The density of reconstructed perforating arteries remained higher with ULM compared to MRI and color Doppler in 6 patients (Figure 5D), indicating the visualization of LSAs with ULM, which is not achievable with conventional imaging modalities.

However, it is interesting to note that the mean intensity of color Doppler in the MCA region is higher in patients in the moyamoya group than in the control group. This effect has already been observed in the literature and categorized as a characteristic pattern of disease progression 6. In this case, ULM would enable us to observe the neoangiogenic structures underlying the phase shift observed on color Doppler.

### ULM comparison between moyamoya and the control group

**Figure 6:**
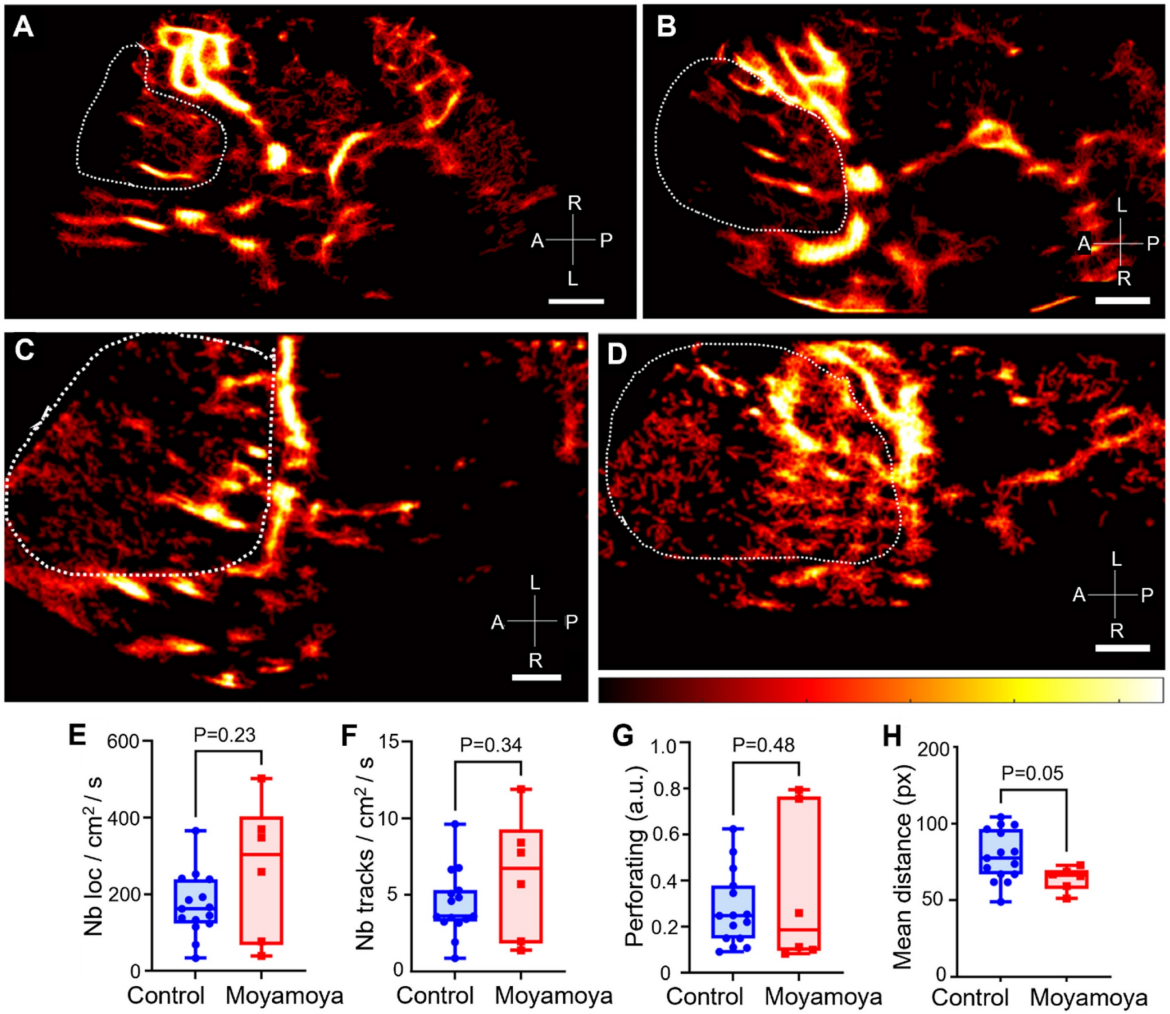
** Comparison of ULM density maps between control and Moyamoya patients.** A) ULM density map of control patient #1 right temporal window, colormap from 0 to 4.5 (a.u.), B) ULM density map of control patients #8 left temporal window, colormap from 0 to 5 (a.u.), C) ULM density map of Moyamoya patient #16 left temporal window, colormap from 0 to 3.5 (a.u.), D) ULM density map of Moyamoya patient #18 left temporal window, colormap from 0 to 2.5 (a.u.). E) Number of localizations per cm² inside perforating arteries' region between Moyamoya and control patients, F) Number of tracks per cm² inside perforating arteries' region between Moyamoya and control patients, G) Number of at least 8 pixels connected objects (a.u.), i.e. perforating arteries, inside perforating arteries' region between Moyamoya and control patients. H) Mean distance between tracks (px) inside perforating arteries' region between Moyamoya and control patients. Same color bar for all patients. White dotted lines indicate perforating arteries' area segmentation. Radiological notation, P: Posterior, A: Anterior, R: Right, L: Left. Student t-tests were performed to compare the two groups (see Methods)

The same regions segmented for comparison of imaging methods, i.e., TOF MRI, ULM, and color Doppler, were reused to compare vessels reconstructed by ULM in the control group (Figure 6A-B) and in the group of patients with moyamoya symptoms (Figures 6 C-D).

Overall, the number of normalized tracks per cm² and the density of microbubble localizations per cm² in the region of perforating arteries showed no significant differences between moyamoya patients and control patients (Figure 6E-F). The number of perforating arteries showed no significant differences between the two groups either (Figure 6G). Finally, the spatial distribution, i.e., mean distance from the average point, of these vessels, calculated here as the mean distance of the tracks from the mean point of all the tracks, tended to be smaller in moyamoya patients than in control ones (P = 0.05, Figure 6H). In control patients, longer and more elongated perforating arteries resulted in a more spread-out distribution.

In moyamoya patients, we were also able to see these dense vessels directly on some arteriography, i.e., the gold standard. Although the 2D slice limits comparison, the supply network could be found on the moyamoya side (patient 4 in Supplementary Figure S9 and in patient 7 in Supplementary Figure S10), but not on the control side (patient 7 Supplementary Figure S10).

## Discussion

### Summary of the entire study

In this study, we demonstrated that 2D transcranial ULM density maps could be obtained with conventional low frame-rate clinical ultrasound scanners. 2D transcranial ULM enabled the reconstruction of 0.8 ± 0.3 mm diameter perforating arteries in the MCA region, perforating arteries that were not visible with other conventional imaging methods, i.e., TOF MRI and color Doppler. In addition, we were able to image part of the vascular network of patients with moyamoya pathology. While color Doppler highlighted pattern differences in these subjects, ULM revealed the morphological differences underlying these phase distinctions.

### Implications

The results of this study are promising regarding the capability of 2D ULM to detect fine cerebral arteries, even with the low frame rate of the acquisitions. Indeed, in addition to the cerebral perfusion that can classically be calculated in CEUS mode, ULM provides information on the layout of the vasculature underlying these vascular dynamics.

Clips from standard clinical ultrasound equipment were utilized in this study. This showcases the method's scalability. Access to publicly available codes further enhances its applicability 28. In this way, it should be easy to explore other organs using the same algorithms coupled with CEUS mode 26.

### Limitations

One of the major limitations of this technique, and more generally of all transcranial ultrasound techniques, is that it can only be applied to patients with an appropriate acoustic window, and we had to exclude 2 patients for this reason. In our study, the average age was relatively low in both groups: 47±14 years in the control group and 43±15 years in the moyamoya group. Among them, 65% of the included patients were men. Nevertheless, it should be noted that, in the literature, around 20% of patients (male and female combined) do not have a sufficient acoustic window for transcranial ultrasound 34.

Another limitation arises from the fact that ULM requires microbubble tracking accumulation at the same position for several minutes, i.e., from 30 seconds to 4 minutes. As the probe was handheld, drift was inevitable. The current solution is to remove these clips from the analyses, which reduces the amount of usable data and, therefore, the number of reconstructed vessels. The ULM algorithm also requires individual microbubbles to be visible in the clips. However, in the case of acquisition in the MCA region, this criterion cannot be met for both large arteries and perforating arteries. Although this limitation has been partially overcome thanks to the selection of upstream clips and the localization of microbubbles using an isolability criterion, i.e., spaced by 5 λ (Supplementary Figures S4, S5, S6), it remains one of ULM's main constraints. Another major limitation of ULM lies in the adaptation of parameters for each patient, i.e., maximum track linking distance and their minimum length. Depending on the visualized phenomena, ULM tracking parameters must be adapted to correctly follow the microbubbles (Supplementary Table 3).

Although we were able to track individual microbubbles on a mesh smaller than the wavelength, i.e., our clips have a pixel size smaller than λ/5, we could not use Fourier Ring correlation (FRC) to measure resolution as in 24 because the ULM-reconstructed vascular system was not sufficiently populated. Our limited frame rate did not allow us to perform a flow pattern analysis as in 10,35,36. Consequently, we provided an estimate of the size of the reconstructed vessels rather than a full measure of resolution. Furthermore, density measurements and the analysis of various vessel sizes rely on manually segmented masks, which introduces subjective bias. Likewise, registration between TOF MRI and 2D ULM is conducted manually by a single radiologist manually, potentially impacting accuracy.

The low frame rate of the acquisitions does not allow us to track the fastest microbubbles within the acquisition, i.e., those above 45.9 mm/sec. In reality, the ULMs produced in this study correspond more to accumulations of locations filtered by the tracking parameter, i.e., the minimum track duration eliminates tracks that are too short. The resulting tracks are short due to the low frame rate of the acquisition, and our mapping is complete thanks to the multiplication of microbubble passages within the different vessels, and, therefore, within the different pixels. It is thanks to this repetition that we are able to morphologically reconstruct the different vessels, each of which is made up of several short tracks. Thus, we focused our metrics on measures of density and distance between tracks rather than on velocity or tortuosity.

Furthermore, acquisitions in CEUS mode did not allow us to recover the raw radiofrequency signals at the output of the ultrasound scanner. As a result, no motion correction 37 or skull-induced aberration correction 38,39,40 could be applied in our case. Nevertheless, many correction techniques exist and could be used to improve ULM reconstruction.

### Perspectives

In the future, high frame-rate ultrasound remains the technique to prioritize. Indeed, the low frame rate imaging as used here has limited us to density reconstruction. Although we were able to distinguish the pathological group from that of the control patients, our analyses and quantifications were based solely on morphological measurements of the vessels. On the other hand, ULM performed with ultrafast ultrasound scanners, i.e., several hundred or even thousands of images per second, could enable measurements of vessel velocities or even pulsatility 41, which is promising for future biomarkers of pathologies. The ultrafast ultrasound scanner could also be useful for estimating and correcting motion to increase the finesse of vessel reconstruction at this scale 37.

Furthermore, volumetric imaging will be essential. 3D ULM has already proven its worth in preclinical studies on numerous organs 16,17,19,23,42,43 and remains indispensable for observing complex vascular structures that are not necessarily aligned in the 2D imaging plane 25. In the case of moyamoya, 3D would enable a detailed description of vessel entanglement due to the pathology's neoangiogenesis. Similarly, in tumors, tracking microbubbles in 3D imaging could potentially enable more precise microvascular reconstruction, avoid false pairing aberrations 25, and allow for finer metric calculations for better therapeutic follow-up 44.

Our study aimed to explore the current potential of transcranial 2D ULM with low frame rate clinical ultrasound scanners and open-source tools so that all hospitals could have access to this technology. Nevertheless, future developments will need to focus on the use of high frame rate ultrasound scanners to enable better tracking of microbubbles and estimation of their displacement velocities. Volumetric acquisitions will also be essential for quantifying complex vascular networks, such as those observed in moyamoya cases. Thus, in the future, high-frame rate 3D ULM could become a new reference technique for research into microvascular diseases.

## Supplementary Material

Supplementary methods and figures.

## Abbreviations

MB: microbubbles
US: ultrasound
ULM: ultrasound localization microscopy
CEUS: contrast-enhanced ultrasound
TOF: MRI time of flight magnetic resonance imaging
LSA: lenticulostriate arteries
MCA: middle cerebral artery
ACA: anterior cerebral artery
PCA: posterior cerebral artery
