# Supplementary material for: Transcranial ultrasound localization microscopy in moyamoya patients using a clinical ultrasound system
Source: Theranostics. 2025 Mar 10;15(9):4074–83. doi: 10.7150/thno.105427 (PMC11980664; doi:10.7150/thno.105427)
Supplement: Supplementary file 1 — Supplementary methods and figures. [file thnov15p4074s1.pdf]

SUPPLEMENTARY MATERIALS

Transcranial Ultrasound Localization Microscopy in moyamoya patients using a clinical ultrasound system

SUPPLEMENTARY METHODS

Transtemporal acoustic window estimation during US acquisitions

The quality of temporal acoustic windows was not defined using quantitative criteria. This information is derived from the clinician's subjective assessment. In some patients, transcranial color Doppler examination enabled visualization of the Circle of Willis and the MCA without the injection of a contrast agent. These patients were considered to have a good acoustic window. Others did not, and the clinician should inject microbubbles to visualize these vessels.

Bolus injection

CEUS acquisition was performed bilaterally on each patient, i.e., 2 mL on each side. In patients with a good acoustic window (as assessed above), 1 mL of Sonovue enabled us to observe single microbubbles in the region of the perforating arteries. We repeated this injection 2 times per temple, waiting visually for the microbubbles to disappear on the ultrasound scanner screen, i.e., the real-time return of the CEUS acquisition, around 2 to 3 minutes after the first injection. In the other patients, we directly injected 2 mL to observe microbubbles in the perforating arteries.

Clips selection by estimating movement and concentration during CEUS acquisitions

To estimate the concentration of microbubbles in the clips, we plotted the intensity of each clip against time. In this way, we were able to select clip numbers corresponding to usable acquisitions with single microbubbles present in the perforating arteries by eliminating excessively noisy acquisitions, i.e., those with a total image standard deviation below the noise threshold which had an empirical value of 2.5 (Supplementary Figure S2).

To estimate the motion within the probe, we calculated a power Doppler per clip by applying a bandpass filter with a frequency cutoff of [2 15] Hz to each clip and summing all the images over time. In this way, we were able to observe the potential drift by correlating the first power Doppler (reference image) with the following ones (Figure S2). Overall, a correlation below 0.9 (arbitrary threshold) represented significant movement (drift or plane change). In the event of a change of plane or excessive drifts, i.e., correlation above the threshold of 0.9, the clips were not selected. It is essential to note that this motion estimation is intimately linked to the concentration estimation, given that we use the same CEUS images. Nevertheless, this step allows us to visually assess the presence of movements. Nevertheless, we were unable to correct motion in our acquisitions as this required retrieving B-mode images in a duplex mode, which reduced the frame rate and therefore our ability to track microbubbles.

Perfusion mask calculation for ULM localization step

A perfusion mask was generated to distinguish microbubble signal and noise. Indeed, the perfusion classically observed in blood vessels has a specific pattern [7,8,9]. Noise, on the other hand, oscillates rapidly with a low standard deviation of intensity. To create this binary perfusion mask, we concatenated all the clip images and performed spatial averaging according to a kernel arbitrarily chosen as 10 x 10 pixels. We then projected the temporal maximum intensity divided by the temporal minimum intensity in each of these kernels (Supplementary Figure S3). This projection was then normalized by the maximum image-wide value of this ratio. Then, the perfusion mask was set to 1 in kernels above the median of this normalized projection ratio and to 0 in those below it. Thus, if the perfusion mask is set to 0, the gap between minimum and maximum intensity is small, indicating a noisy zone. Conversely, the classic vascular pattern results in a strong maximum and a weak minimum, generating regions of the perfusion mask set to 1. This binary mask was then used during the ULM localization step (Figure S2).

PSF analysis for ULM localization step

The localization step is crucial in ULM, as it will largely determine which vessels can be reconstructed in the final density mapping. In the case of localization in the MCA region, microbubbles tend to be highly concentrated in the large vessels (Circle of Willis and middle cerebral artery), while they are more isolated in the perforating arteries. To localize microbubbles in these different arteries simultaneously (MCA and perforating arteries), we considered microbubbles "close together" at 2 λ (10 pixels) and more "isolated" microbubbles spaced 5 λ apart (30 pixels). In the 15 control patients, we independently reconstructed the ULM density mappings by performing a single localization of isolated microbubbles (Figure S3 A) and of closely spaced microbubbles (Figure S3 B). We were thus able to quantify that the localization of microbubbles spaced 2 λ apart enhanced the MCA region, while the localization of microbubbles spaced 5 λ apart enhanced the region of the perforating arteries (Figure S3 C,D). We therefore opted for dual localization to enhance the reconstruction of both the MCA and perforating arteries (Figure S3 E).

In ultrasound, the lateral resolution of a point source evolves with depth according to the following relationship: $Res_{Lat} = \frac{\lambda z}{D}$, with λ the probe wavelength, i.e., 0.77 mm, z the depth and D the probe aperture, i.e. 16.3 mm. The spot of a microbubble, i.e., the PSF, close to the probe will therefore be less spread out than the spot of a microbubble at depth. To include this PSF evolution, we considered that the σ of the PSF sought during the ULM localization stage would evolve as a function of depth, i.e., a σ of 1 λ at a depth of less than 3 λ (2mm) up to a σ of 3 λ at depths greater than 70 λ (5 cm). In Figure SF5 we compare ULM mapping reconstructed with a PSF of 30 edge pixels (5 λ) and a σ of 7 pixels (1 λ) (Figure S5 A), or with a σ of 25 pixels (4 λ) (Figure S5 B) or with a depth-adapted σ, i.e. from 5 (1 λ) to 14 pixels (3 λ) (Figure S5 C). The evolution of the average intensity of these depth maps confirms that adapting the size of the σ to depth enhances both high and low perforating arteries (Figure S5 D).

Control patient n°	Age	Sex	Stroke side on MRI	Thrombectomy	Thrombolysis	Stroke etiology	Included
1	35	M	Left MCA	no	yes	cardioembolic, FOP	yes
2	57	M	Right MCA	no	no	atheroesclerosez, estenoses carotide	yes
3	65	M	Right cerebellum	no	yes	atherosclerosis, occlusion AVD	yes
4	75	M	Left MCA	no	no	atherosclerosis, carotid stenosis	yes
5	46	M	Left MCA	no	no	cardioembolic FA	yes
6	47	M	Left MCA	no	yes	prothrombotic post heavy digestive surgery	yes
7	51	M	Left PCA	no	yes	cardioembolic, EF 40% apical akinesia	yes
8	50	M	Bilateral PICA	no	no	unknown	yes
9	50	M	Right PICA	no	no	unknown	yes
10	43	M	Left MCA	no	no	unknown	yes
11	31	M	Right MCA	yes	yes	cardioembolic, FA	yes
12	31	W	Right pontic area	no	no	unknown	yes
13	33	W	Left cerebellum Right frontal area	no	no	cardioembolic, FOP	yes
14	66	M	Right MCA	no	no	unknown	yes
15	31	W	Left MCA	no	yes	cardioembolic, FOP	yes

Moya Patient n°	Age	Sex	Moya side on arteriography	Network description	Included
16	50	M	Bilateral	Near occlusion termination bilateral carotid with collateral network	yes
17	43	W	Bilateral	Moderate Moya network	no
18	28	M	Left	Overdevelopment of lenticulostriate arteries in MCA left territory	yes
19	47	M	Right	Right carotid end stenosis and right MCA occlusion with collateral network	yes
20	60	W	Right	Right unilateral Moya	yes
21	30	M	Left	Unilateral left moya network	yes
22	20	M	Right	Unilateral Moya in the region of right MCA	yes
23	57	W	Bilateral	Bilateral Moyamoya	no
24	56	W	Left	Right central posterior parasagittal meningioma. Moyamoya of right carotid termination. Left ACM co-post developed.	no

Supplementary Table *S1. Clinical characteristics of patients (FA: auricular fibrillation, FOP: permeable oval foramen, AVD: distal venous arterialization, EF: left ventricular ejection fraction)*

Patient n°	Temporal Window side	CEUS duration (sec)	ULM perforating area (cm²)	Doppler perforating area (cm²)	MRI perforating area (cm²)
1	Right	107	6,4	9,5	16,2
2	Left	46	6,1	6,9	6,9
3	Left	49	4,2	8,9	6,6
4	Right	59	9,4	6,1	7,6
5	Right	173	8,7	2	12,9
6	Right	91	11,7	5,7	4,2
7	Right	80	8,3	4,9	13,2
8	Left	90	6,2	3,9	9,4
9	Left	58	5,4	5,3	9,1
10	Left	35	9,3	7,7	10,8
11	Right	30	6,2	4,5	7,5
12	Left	86	7,9	5	10,9
13	Right	61	9,8	6,4	18
14	Right	176	7,4	6,3	6,2
15	Right	182	7,7	5,8	7,8
16	Left	230	8,8	5,3	6,6
17	Registration error as assessed on motion estimation				
18	Left	30	9,9	9,1	6,3
19	Right	161	5,6	11,6	7,5
20	Right	187	6,6	10,5	5,2
21	Left	108	5,7	4,2	11,1
22	Right	31	7,2	8,9	7,2
23	Poor temporal window as assessed by preliminary transcranial Doppler				
24	Poor temporal window as assessed by preliminary transcranial Doppler				

Supplementary Table S2*. Acquisition parameters and segmented areas*

	**PSF 10-pixels**			**PSF 30-pixels**		
**Patient n°**	**max linking distance (λ)**	**mag gap closing (frames)**	**min length (frames)**	**max linking distance (λ)**	**mag gap closing (frames)**	**min length (frames)**
**all patients for metrics**	2	1	2	3	1	2
**display P1**	3	1	2	3	1	2
**display P8**	2	1	2	3	1	2
**display P10**	3	1	2	3	1	2
**display P16**	2	1	2	2	1	2
**display P18**	2	1	2	2	1	2
**display P20**	2	1	2	2	1	2
**display P22**	2	1	2	2	1	2

Supplementary Table S3*. ULM parameters for metrics calculation (all patients), and for display of the different figures in this article.*

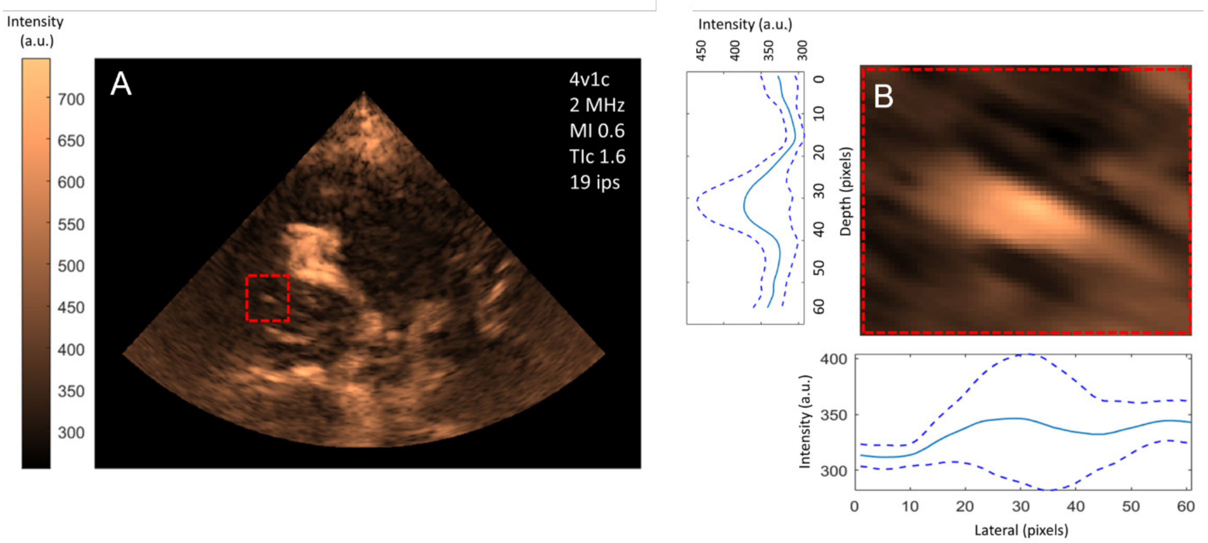

Supplementary Figure S1. A) CEUS acquisition and parameters, and B) example of microbubble size estimation at 5 cm depth. *By measuring the full width at half maximum, we get a round value of 14 pixels (i.e. 1.8 mm) for the PSF of the microbubble localized at 5 cm depth. This estimation has a good correlation with the theoretical value of the lateral resolution at 5 cm depth, i.e. 2mm with a 2 MHz probe and a 19.2 mm aperture.*

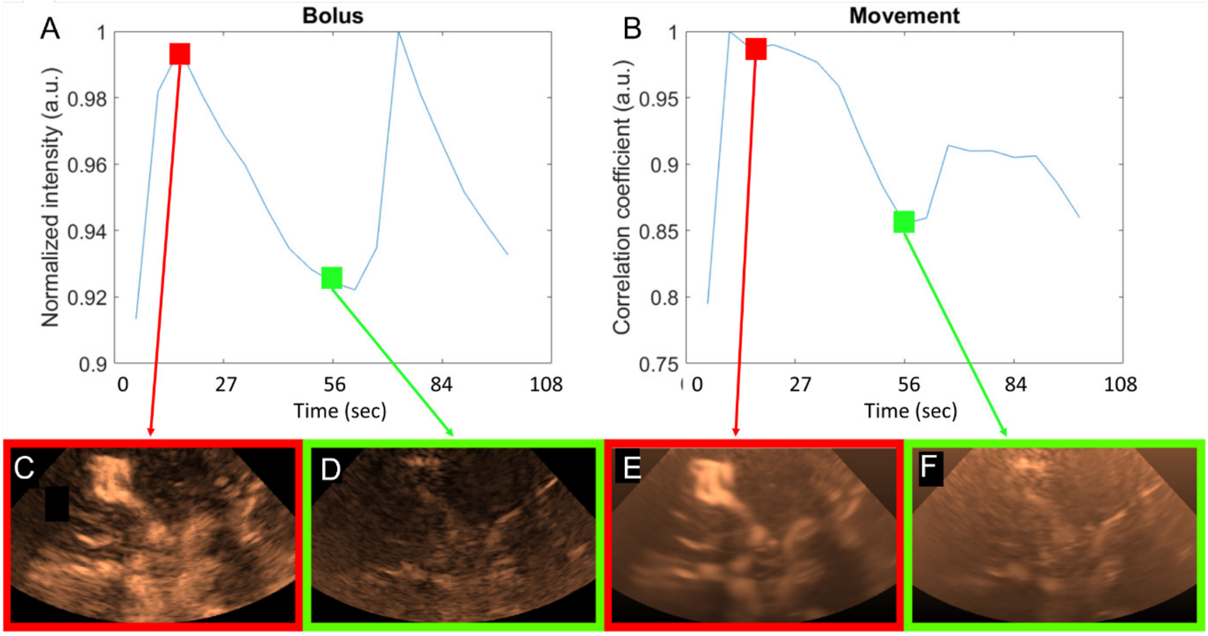

Supplementary Figure S2. Estimation of MB concentration and probe movements in patient 1. A) *Concentration study: value of mean intensity normalized as a function of clip number, B) Movement study: correlation coefficient between the power Doppler of clip 2 and the power Doppler of the other clips, C) Peak of the first bolus (image from clip 2), D) End of first bolus (image from clip 10), E) Power Doppler of clip 2, f) Power Doppler of clip 10: a slight rightward drift can be observed.*

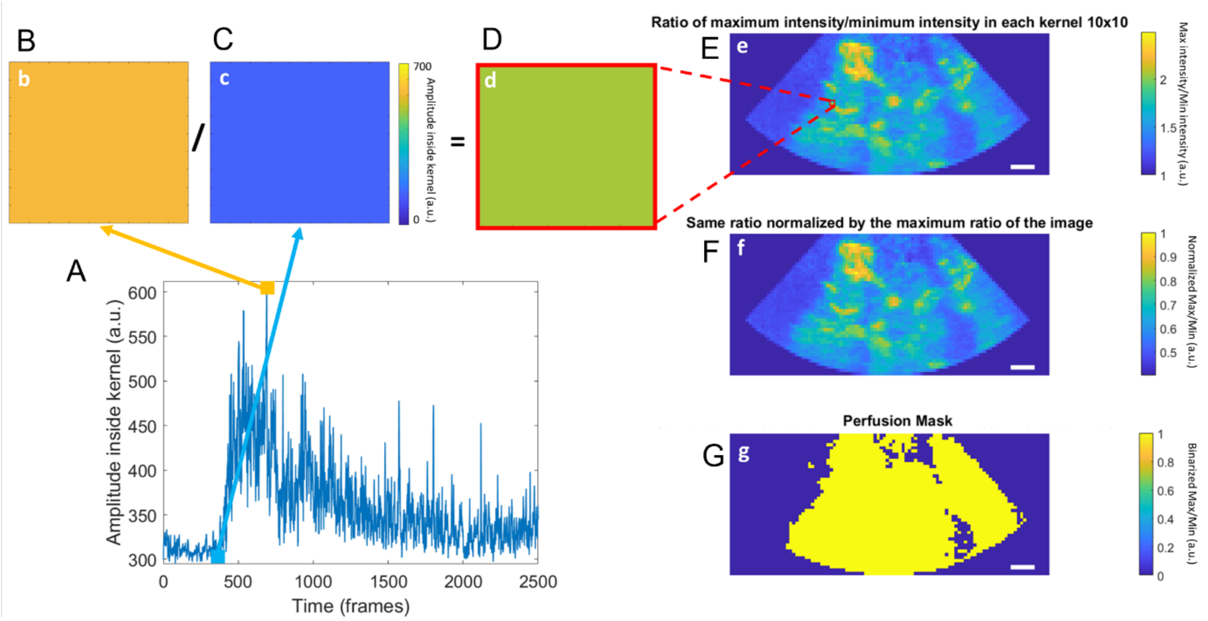

Supplementary Figure S3. Creation of a perfusion mask to avoid localizing noise. *A) Perfusion curve (intensity vs. time) within the 10x10-pixel kernel zoomed in e), B) Maximum intensity of this 10x10-pixel kernel, C) Minimum intensity of this 10x10-pixel kernel, D) Result of dividing this kernel (maximum over minimum) zoomed in E), e) Maximum temporal intensity divided by the minimum temporal intensity in each of the 10x10 pixel kernels. The kernel shown in b, c, and d is framed in red, F) the Same ratio normalized by the maximum value, in the whole image, of this ratio, G) the Perfusion mask corresponding to this normalized ratio and binarized with a fixed threshold (i.e. the median of the normalized ratio of the image presented in f). 1cm scales.*

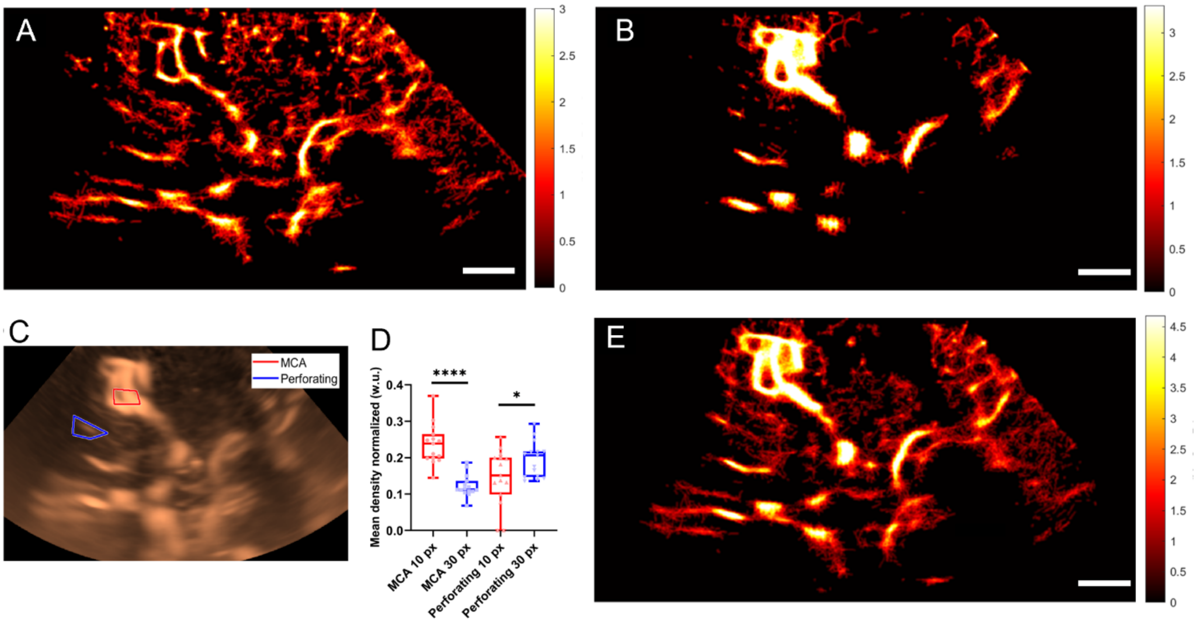

Supplementary Figure S4. Influence of the size of the PSF on ULM density maps: the necessity of the double localization step. Study of 10 pixels (2 λ) PSF and 30 pixels (5 λ) PSF locations in the 15 control patients. *A) Vascular density mapping using the 30 pixels PSF criterion in patient n°1. Colormap in arbitrary units (a.u.), scale 1cm, B) Same for 10 pixels PSF locations, C) Segmentation of MCA and perforating on patient n°1, D) Comparison of intensity in each of the regions (MCA and perforating) on both 10 and 30 pixels PSF mapping in the 15 control patients. Student's t-test to compare MCA and perforating, E) Vascular density mapping performed by merging the two fusions of the two locations in patient n°1. ULM density maps are displayed in arbitrary units and are displayed with a square compression and a gaussian smooth filter.*

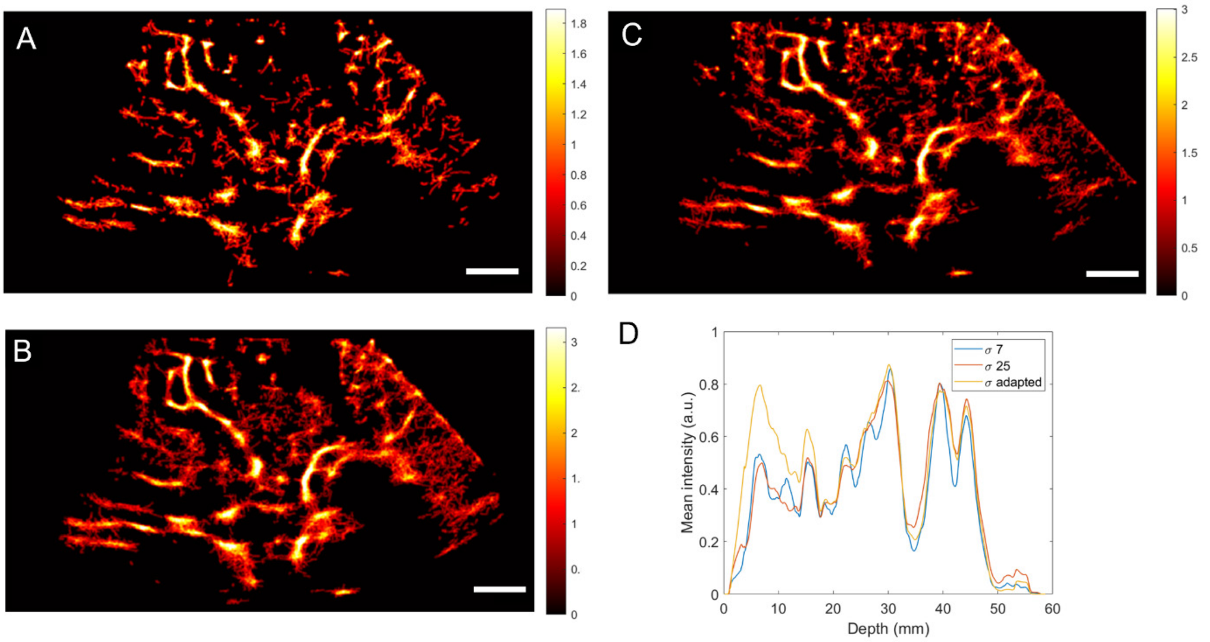

Supplementary Figure S5. Influence of the σ of the PSF on ULM density maps: the necessity of the adapted σ as a function of depth. *A) For a PSF size set at 30 pixels (isolated), the standard deviation of the Gaussian PSF σ set at 7 seems to consider microbubbles too isolated, and therefore tends to create holes in the mappings, B) for the same PSF size using a σ of 25 pixels enhances vessels in-depth, C) for the same PSF size, the σ adapted in-depth seems to enhance low and high perforating arteries, which is confirmed by the intensity analysis of density maps as a function of depth D). Colormap in arbitrary units (a.u.), scale 1cm. ULM density maps are displayed in arbitrary units and are displayed with a square compression and a gaussian smooth filter.*

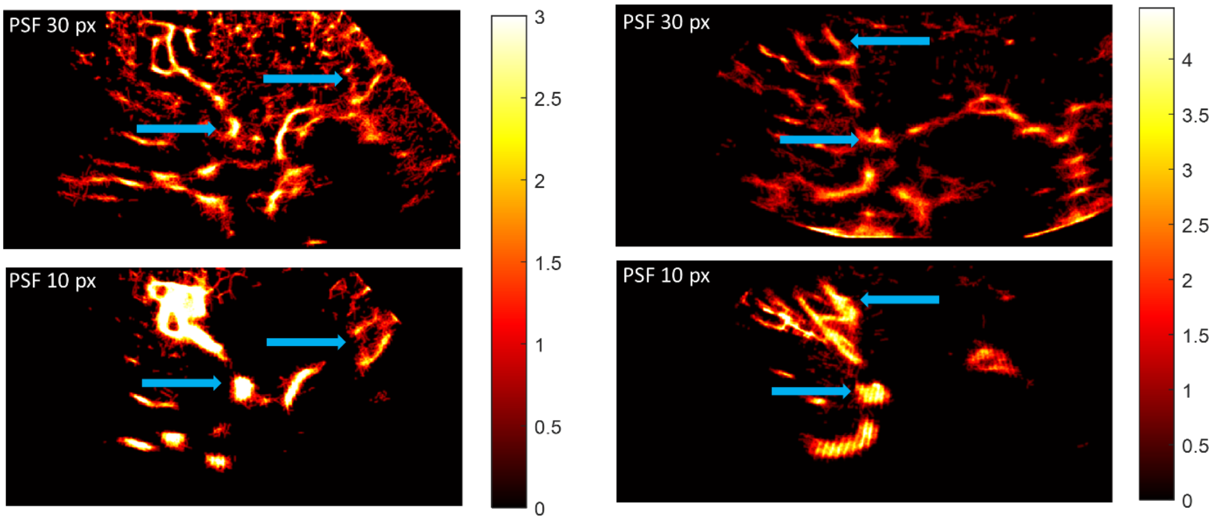

Supplementary Figure S6. Utility of a combined localization step to localize the whole vasculature. *The localization of a PSF with 30 pixels will lead to the reconstruction of small isolated vessels. Whereas the localization of a 10 pixels PSF will lead to the bigger concentrated vessels reconstruction. Patient 1 on the left show that 10-pixel PSF localizations lead to a better estimation in the carotid and in the posterior cerebral artery than with the 30-pixel PSF localizations (blue arrows). Similar conclusions were found on other patients, such as patient 8 (right), with a better reconstruction of the second segment of the middle cerebral artery and of the carotid (blue arrows) with a 10-pixels PSF. ULM density maps are displayed in arbitrary units and are displayed with a square compression and a gaussian smooth filter.*

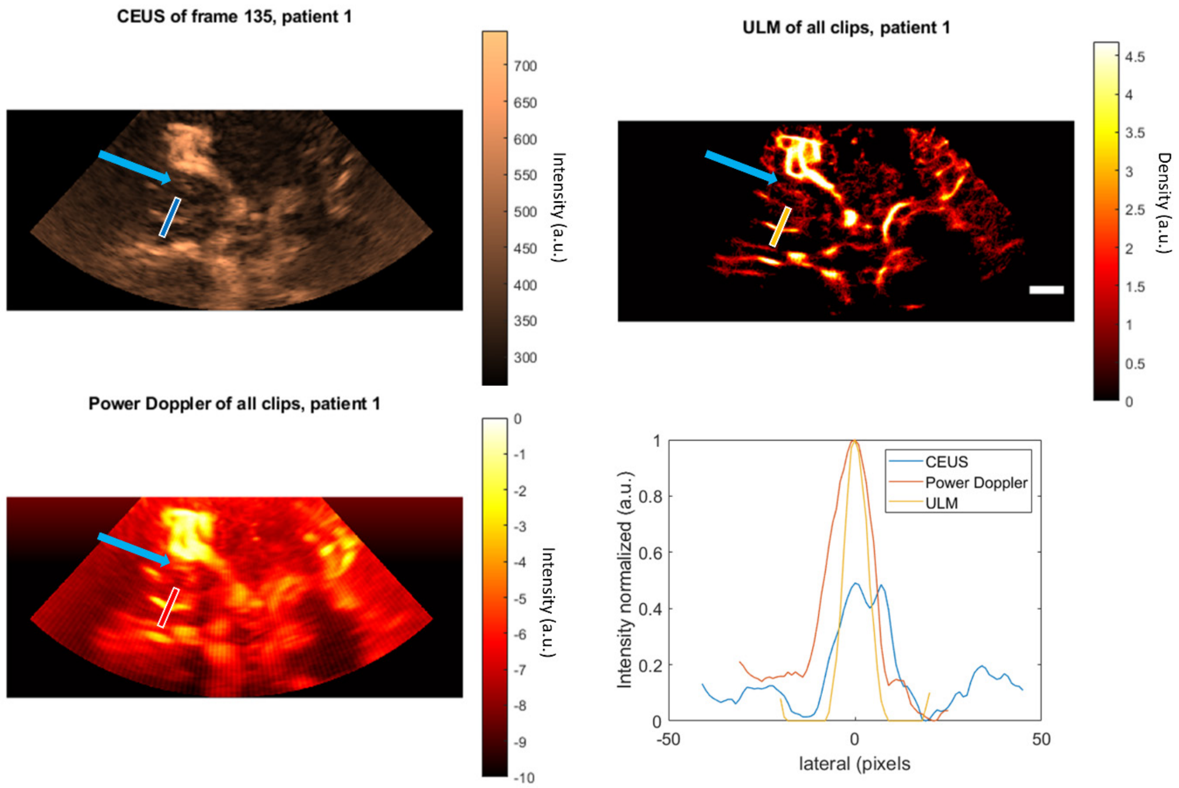

Supplementary Figure S7. Comparison of cross section of 1 perforating artery between CEUS single frame, ULM density map and cumulated frame over time, i.e. Power Doppler. *The estimation of the perforating artery by cross section and FWHM measurement show that ULM has the finest vessel reconstruction compared to Power Doppler and CEUS (1 mm, 1.75 mm and 2mm respecively). The CEUS single frame (upper left) does not enable all vessel reconstructions (blue arrow) even if some of them are visible due to high concentration of microbubbles at the beginning of the bolus. ULM density map is displayed in arbitrary units and are displayed with a square compression and a gaussian smooth filter.*

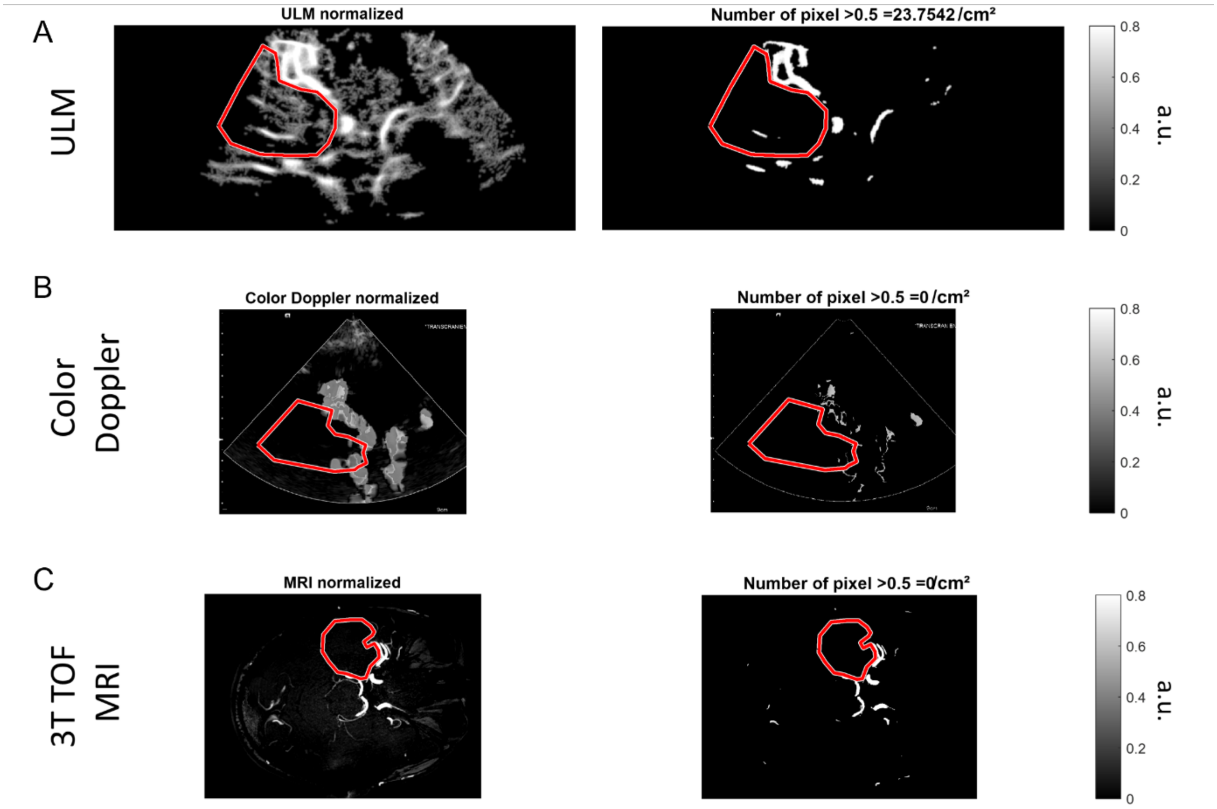

Supplementary Figure S8. Example of pixel vessel density counting in patient 1. *A) In ULM, B) Color Doppler and C) 3T TOF MRI. The images were normalized and only pixel > 0.5 were considered as vessels. These number of pixels considered as vessels were normalized per cm² of the perforating arteries area (highlighted with a red mask).*

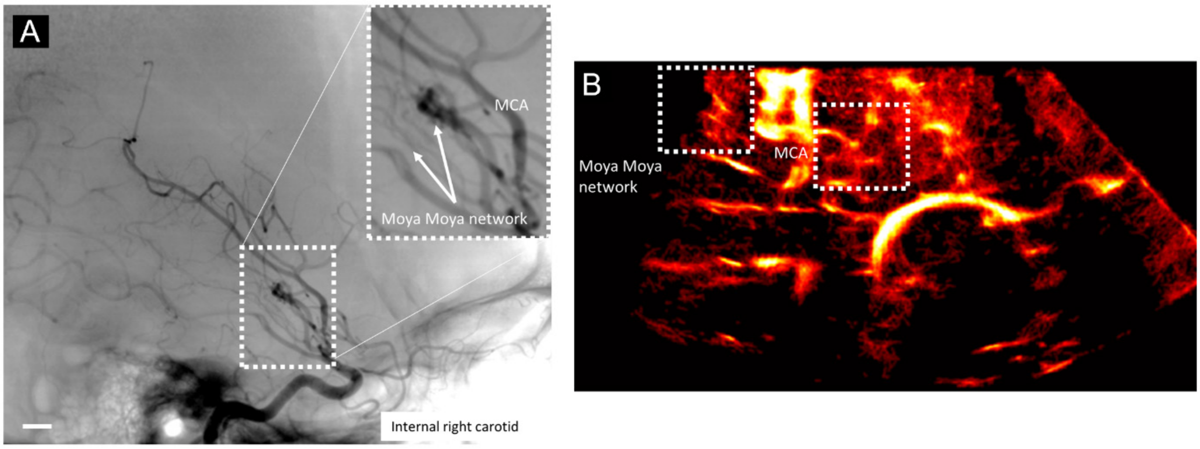

Supplementary Figure S9. Comparison between arteriography and ULM on a Moyamoya case, patient 20. *A) Time capture of intra-arterial angiography acquisition via right catheterization. Sagittal plane, 1cm scale. The white dotted square shows the zoomed area where we can identify collateral Moyamoya network and Middle Cerebral Artery (MCA). B) Transtemporal right side ULM density map of the same Moyamoya patient. White dotted squares indicate the presence of the Moyamoya supply network. We can also identify the MCA in the ULM density map. ULM density map is displayed in arbitrary units and are displayed with a square compression and a gaussian smooth filter.*

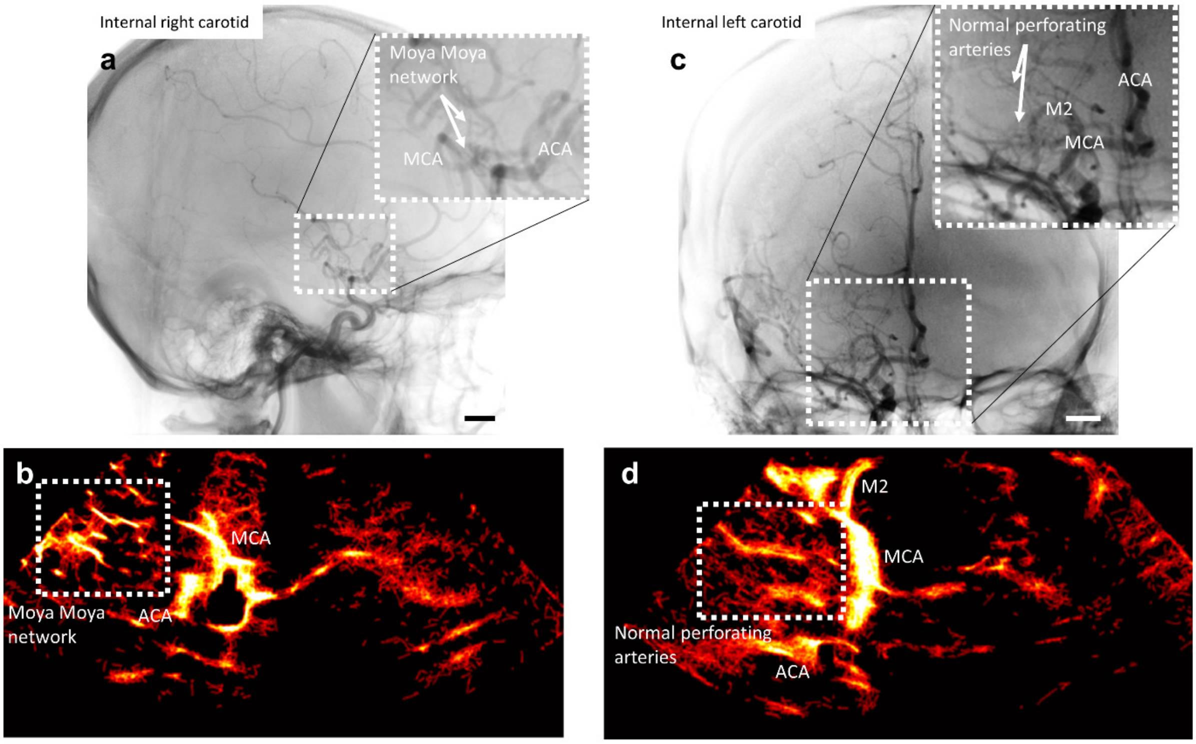

Supplementary Figure S10. Comparison between arteriography and ULM on Moyamoya unilateral case (right), patient 22. *a) Time capture of intra-arterial angiography acquisition via right carotid catheterization.. Sagittal plane, 1cm scale. b) Transtemporal right side ULM density map of the same Moyamoya patient. White dotted circles indicate the presence of the Moyamoya supply network in the two imaging modalities. c) Time capture of intra-arterial angiography acquisition via left carotid catheterization. Coronal plane, 1cm scale. d) Transtemporal ULM left side. ULM density maps are displayed in arbitrary units and are displayed with a square compression and a gaussian smooth filter.*
